# Supplementary material for: FERN – a Java framework for stochastic simulation and evaluation of reaction networks
Source: BMC Bioinformatics. 2008 Aug 29;9:356. doi: 10.1186/1471-2105-9-356 (PMC2553347; doi:10.1186/1471-2105-9-356)
Supplement: Additional file 1 — FERN distribution, Version 1.3. This archive contains the FERN source code and binaries as well as documentation and example models in FernML and SBML. [file 1471-2105-9-356-S1.zip › fern/doc/javadoc/fern/simulation/algorithm/package-summary.html]

fern.simulation.algorithm


---


|  |  |  |
| --- | --- | --- |
| **Overview** \| **Package** \| Class \| **Use** \| **Tree** \| **Deprecated** \| **Index** \| **Help** | |  |
| **PREV PACKAGE**   **NEXT PACKAGE** | **FRAMES**    **NO FRAMES**     **All Classes** |  |


---

## Package fern.simulation.algorithm

Provides algorithms for stochastic simultion of reaction networks.

**See:**
  
          **Description**

| **Class Summary** | |
| --- | --- |
| **AbstractBaseTauLeaping** | Base class for all tau leaping procedures (which are different in the methods choosing the timestep candidates for critical and noncritical reactions). |
| **AbstractTauLeapingPropensityBoundSimulator** | There are some possibilities to bind the expected change of the propensities by a value epsilon in order to fulfill the leap condition. |
| **DependencyGraph** | Implements a dependency graph. |
| **GibsonBruckSimulator** | Implementation of the algorithm of Gibson and Bruck. |
| **GillespieEnhanced** | This is an enhanced version of the original Direct method developed by Gillespie. |
| **GillespieSimple** | Implementation of Gillespie's Direct method. |
| **HybridMaximalTimeStep** | This is an modified version of Maximal Time Step Method by Puchalka and Kierzec. |
| **IndexedPriorityQueue** | Implements an indexed priority queue. |
| **TauLeapingAbsoluteBoundSimulator** | There are some possibilities to bind the expected change of the propensities by a value epsilon in order to fulfill the leap condition. |
| **TauLeapingRelativeBoundSimulator** | There are some possibilities to bind the expected change of the propensities by a value epsilon in order to fulfill the leap condition. |
| **TauLeapingSpeciesPopulationBoundSimulator** | There are some possibilities to bind the expected change of the propensities by a value epsilon in order to fulfill the leap condition. |

## Package fern.simulation.algorithm Description

Provides algorithms for stochastic simultion of reaction networks.

---


|  |  |  |
| --- | --- | --- |
| **Overview** \| **Package** \| Class \| **Use** \| **Tree** \| **Deprecated** \| **Index** \| **Help** | |  |
| **PREV PACKAGE**   **NEXT PACKAGE** | **FRAMES**    **NO FRAMES**     **All Classes** |  |


---
